# Determinants of prescribing decisions for off-patent biological medicines in Belgium: a qualitative study

**DOI:** 10.1186/s12913-022-08591-1

**Published:** 2022-09-29

**Authors:** Yannick Vandenplas, Steven Simoens, Philippe Van Wilder, Arnold G. Vulto, Florian Turk, Isabelle Huys

**Affiliations:** 1 Department of Pharmaceutical and Pharmacological Sciences, KU Leuven, Leuven, Belgium Germany; 2 Ecole de Santé Publique, Université Libre de Bruxelles (ULB), Brussels, Belgium Germany; 3 Hospital Pharmacy, Erasmus University Medical Center, Rotterdam, the Netherlands; 4 Unversity of Paderborn, Paderborn, Germany

**Keywords:** Biological medicine, Biosimilar, Policy, Behavior, Prescription, Belgium

## Abstract

**Background:**

A competitive market for off-patent biologicals leads to more affordable and high-quality healthcare. In recent years, Belgium has been characterized by its low use of biosimilars and by its shifts from off-patent biologicals toward new alternative therapies. Yet, the prescribing decisions involved in these observations are poorly understood. This study aims to better understand prescribing choices among Belgian physicians in the ambulatory care setting.

**Methods:**

This study consisted of two phases. First, a scoping literature review to identify determinants of prescribing choices was conducted. Scientific databases (Embase and PubMed) were searched until 4 November 2021. Second, the nominal group technique (NGT) was employed during focus group discussions with Belgian physicians to consider and validate these determinants for off-patent biologicals in the Belgian context. The qualitative data resulting from the literature review and focus group discussions were analyzed using the thematic framework method.

**Results:**

Fifty-three scientific articles that discussed elements that determine prescribing choices were identified. Out of these, 17 determinants of prescribing choices were found. These were divided into five categories: (1) product-related, (2) physicians’ personal, (3) healthcare system-related, (4) patient-related, and (5) determinants related to the pharmaceutical company or brand. Nineteen Belgian physicians from different therapeutic areas that regularly prescribe biologicals then participated in focus group discussions. Using the NGT, the group discussions revealed that prescribing choices for off-patent biologicals are determined by a complex set of elements. Clinical data, geographical region, working environment, pharmaceutical marketing, patient profile, clinical guidelines, and preference of key opinion leaders (KOL) were considered most influential. Physicians indicated that the importance of these determinants differs depending on product classes or therapeutic domain.

**Conclusions:**

Multiple elements determine the choice of an off-patent biological or biosimilar product. The importance of each of these determinants varies depending on the context in which the prescribing choice is made. To increase the prescription of best-value biologicals in the Belgian ambulatory care, a set of synergistic measures is required including information for healthcare providers (HCP) and patients, prescribing feedback, prescribing targets, tangible incentives, KOL involvement, guidelines regarding pharmaceutical promotion, and regular revision of reimbursement modalities.

**Supplementary Information:**

The online version contains supplementary material available at 10.1186/s12913-022-08591-1.

## Background

Governments have increasingly experienced difficulties in keeping their healthcare budgets under control over the past few decades. This problem has become even more pressing as there is a growing number of challenges faced by healthcare systems worldwide, such as ageing populations, the emergence of expensive innovative therapies like precision medicine, and the recent COVID-19 pandemic [1]. Belgium is no exception and faces these challenges as well. Belgian healthcare spending has increased annually and is now among the highest in the European Union [2]. A significant portion of healthcare spending is on pharmaceuticals [3]. The most recent data show that pharmaceuticals represent 18% of the total Belgian healthcare budget, with an overall pharmaceutical budget of approximately 5 billion euros in 2021 [4–6]. Moreover, the pharmaceutical budget in Belgium has risen rapidly in recent years, with a growth of 10.7% in 2021. This situation is no longer sustainable given the limited resources available in a country where healthcare is mainly publicly funded. The national health insurer (the National Institute of Health and Disability Insurance or NIHDI) also identified this problem during their recent budget discussions [4, 7].

Therefore, there is a clear need to develop strategies to maintain the financial sustainability of the Belgian healthcare system. One of these strategies should focus on the rational and cost-effective use of medicines, more specifically of biological medicines. Biological medicines or *biologicals* are medicines produced by living organisms, such as bacteria or animal cells [8]. They have been successful in treating several life-threatening or chronic disorders such as cancer, diabetes, and immune mediated inflammatory disorders. Because of a costly development and manufacturing process, biological medicines are generally more expensive compared to traditional chemical molecules [9]. Together with their widespread use, this results in biologicals representing a high cost to national healthcare systems. To date, biologicals represent about 30% of all pharmaceutical spending across Europe [5]. The expected advent of a large number of new biological therapies in the coming years will probably result in a further increase of this share [1]. After the expiry of market exclusivities of biologicals and the market entry of similar versions of authorized biologicals (i.e., biosimilars), off-patent biologicals obtain a more favorable cost-effectiveness profile [10]. Yet, in order to benefit from these more cost-effective medicines, a sustainable off-patent biologicals market is required. A sustainable market refers to a situation in which both off-patent biological and biosimilar products can coexist. It therefore not only strives towards a competitive market, but also towards economic viability for pharmaceutical industry [11]. However, it has become clear that the Belgian market is not sustainable [12, 13]. Significant shifts from off-patent biologicals towards new therapeutic classes (such as interleukin (IL) inhibitors, Janus Kinase (JAK) inhibitors, etc.) and the dominant market position of originator biologicals that lost their market exclusivities (and thus a low use of biosimilars) are the main elements supporting this observation [12–14]. Biosimilars (including etanercept, adalimumab, and insulin glargine) have been part of Belgium’s ambulatory care since 2016. Yet, their market shares have only increased slowly over the past few years and remain very limited [12, 13]. This is especially so in the ambulatory segment of the market where the choice for a particular biological is made by the prescribing physician and is not subject to tender decisions [12, 13].

Due to substantial mandatory price reductions for both biosimilars and reference biological products, price differences are limited and both generate savings for the Belgian healthcare system. Therefore, the concept of best-value biologicals was introduced, which recognizes that both originator biologicals and biosimilars can contribute to a more sustainable healthcare system [13, 15]. The Belgian government supports the concept of best-value biologicals and introduced several initiatives to encourage the use of cost-effective biologicals in the past years [12]. In addition, given the very low biosimilar market shares in Belgium’s ambulatory care, the Belgian government has aimed to specifically increase the use of biosimilars as well [4, 12, 16, 17]. However, these initiatives have not been successful to date, underlining a poor understanding of what drives prescribing decisions within the off-patent biologicals market [12, 13]. As a result, it is of particular interest to better understand what elements determine the choice between different molecules, as well as between a reference biological and its biosimilar(s). In order to design policy interventions to stimulate physicians to prescribe cost-effective medicines in the future, the underlying drivers behind prescribing decisions have to be clearly understood [18, 19].

This study aimed to identify elements that determine the prescription choice for off-patent biologicals in the Belgian ambulatory setting. In doing so, we must not lose sight of the current reality and set the goal of looking beyond the choice between reference products and their biosimilar(s). As described earlier, when choosing a biological product, new molecules or follow-on products are also considered. Ultimately, a better understanding of prescribing choices can be used to inform Belgian policymakers when they are designing interventions to stimulate the prescription of best-value biological medicines or biosimilars.

## Methods

**Fig. 1:**
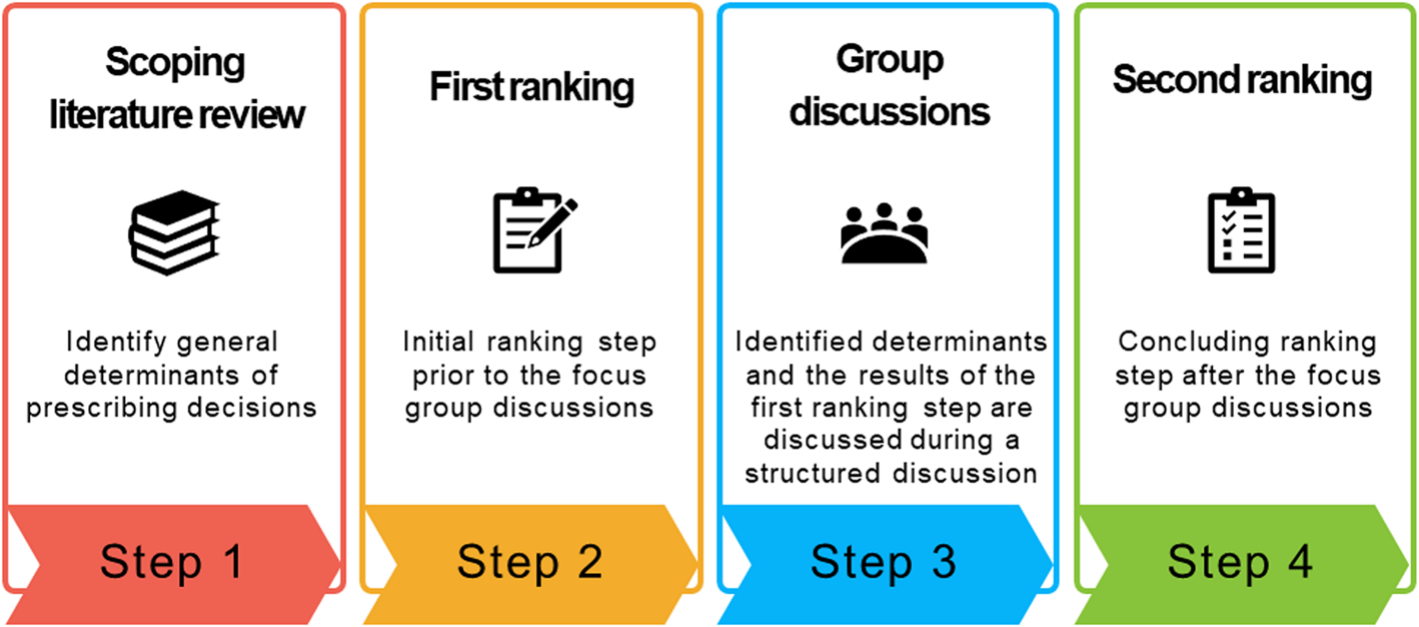
Overview of the methodological approach of this study

Since there currently is no published evidence on the kinds of elements that determine the prescription of off-patent biological medicines, a two-step qualitative approach was used to identify these determinants. During the first step, an overview was made of elements that generally determine prescribing behavior based on a scoping literature review of scientific databases. This served to inform the next step where these identified elements were discussed in focus groups with Belgian physicians. An overview of the consecutive steps of the used methodological approach is provided in Fig. 1.

### Scoping literature review

A scoping review of the literature in Embase and PubMed (MEDLINE) was conducted using a predefined search strategy. Scoping literature reviews aim to identify and map the existing evidence related to a certain topic of interest in a systematic way [20, 21]. Full-text, peer-reviewed, English articles that were published between 2008 and 2021 were eligible for inclusion. Both original research articles and literature reviews were considered. Only articles that described determinants of prescribing decisions were included. Search terms were therefore related to ‘prescribing’, ‘behavior’, and ‘physician’. These search terms were adjusted according to the respective scientific database. Initially, the literature was searched up to March 2021 but was subsequently updated to 4 November 2021. The full literature search protocol can be found in the Supplementary Material.

All identified articles were imported into Mendeley software and duplicates were removed. The remaining articles were then screened by one reviewer (YV) based on title and abstract using Rayyan software [22]. Subsequent screening based on the full text of the selected articles was conducted by the same researcher (YV), and the remaining articles were included in the qualitative analysis. Peer-reviewed articles describing determinants that influence prescribing choices were analyzed qualitatively using the thematic framework method described by Gale et al. [23]. This process included mostly inductive coding as the researchers did not know in advance which determinants were involved in prescribing decisions. During the analysis of the first articles by two researchers (YV and IH), similar codes were identified as determinants of prescribing behavior and were grouped together to form a coding tree (i.e., thematic framework). The codes of the developed framework were agreed upon by the two researchers who coded the first articles. Subsequently, the remaining articles were analyzed by placing relevant passages under the developed thematic framework. In case new themes or codes were found, these were added to the existing framework. In the last phase, the results were summarized in a framework matrix and interpreted by the researchers.

### Focus group discussions using the nominal group technique (NGT)

#### Conduct

After general determinants of prescribing decisions were identified from the scoping literature review, the purpose of this study was to further examine these determinants in relation to off-patent biological medicines in the Belgian context. The results of the scoping literature review were therefore used as a starting point. Each of the identified determinants were discussed separately during focus group discussions and were scored quantitatively by the participants according to their relative importance. The nominal group technique (NGT) was used to achieve the latter. The NGT is a method to establish structured group discussions or interactions, which encourages all participants to provide their insights and to react to these insights [24]. It was chosen since the NGT allows for structured group discussions with the purpose to achieve an agreement related to a particular topic of interest. The NGT was considered appropriate for this research question as the technique also allows for ranking of priorities and the generation of new ideas [24].

The NGT consists of four distinct parts as defined by Delbecq and Van de Ven: (i) silent generation, (ii) round robin (i.e., the moderator asks each participant to share a single idea with the group), (iii) clarification, and (iv) ranking [24, 25]. However, the NGT is a highly adaptable method depending on the specific research question and desired outcomes [24]. This allows researchers to replace or skip certain steps. For example, in this study, silent generation was omitted and replaced by a comprehensive literature review. Based on the ideas identified (in this case, determinants of prescribing behavior), the participants performed an initial ranking step prior to the group discussions. During the ranking exercise, each participant was asked to score the importance of each of the identified determinants on a five-point Likert scale in the following way: 1 = very low impact, 2 = low impact, 3 = some impact, 4 = high impact, 5 = very high impact. Average ranking values were calculated per determinant. In addition, participants were asked if there were other determinants influencing prescribing decisions regarding off-patent biologicals or if certain elements were irrelevant in this regard. After the initial ranking step, the traditional steps of the NGT were followed. As a result, the NGT consisted of the following steps:

(i) First grading step prior to the group discussion, based on the list of determinants of prescribing decisions resulting from the scoping literature review
(ii) Presentation of the aggregated results of the first grading step to the participants (i.e., average ranking values per determinant)
(iii) Group discussion where each participant was asked to comment on the identified determinants of prescribing decisions and the results of the first grading step
(iv) Second grading step where participants were asked to rank the identified determinants again
(v) Concluding step where each participant could share any final thoughts or remarks.

Usually, group discussions take place face-to-face, but the COVID-19 pandemic situation did not allow for physical interactions at the time when the study was conducted. Therefore, all the group discussions took place remotely via Microsoft Teams. The focus group discussions were anticipated to last between 90 and 120 minutes. All focus groups were moderated by the same researchers (YV and IH), who have experience with conducting qualitative research.

#### Participant selection and recruitment

Participants were Belgian specialist physicians working in therapeutic domains where off-patent biologicals and biosimilars are being prescribed in the ambulatory care setting. These products are listed in the Supplementary Material and mainly include tumor necrosis factor (TNF)-alpha inhibitors and insulin analogues. Therefore, eligible participating physicians were endocrinologists, dermatologists, gastroenterologists, and rheumatologists. The physicians were required to have a good knowledge of biologics and experience with prescribing both originator biologicals and biosimilars. The physicians were selected based on purposeful sampling, that is the identification and selection of individuals or groups who are especially knowledgeable about the topic of interest [26]. Participants were contacted via e-mail. Their contact details were obtained via publicly available information or through the network of the research unit where this study was conducted.

NGT group discussions usually involve two to fourteen participants, with an ideal number of seven participants [24]. To ensure a good balance between input coming from a variety of participants and giving each participant sufficient time to speak, the researchers aimed to have a minimum of four and a maximum of ten participants per group. The researchers ensured that a variety of backgrounds (i.e., region, working environment, therapeutic area) were present in each of the focus groups. Differences in language (i.e., Dutch or French) were also considered. Therefore, participants were asked to speak English during the focus groups. However, if necessary, participants could express themselves in their native language and the moderators acted as translators. Eligible participants were invited via e-mail and provided with all relevant study information and the informed consent form (Cfr. Supplementary Material). All participants were required to provide written informed consent prior via e-mail to the start of the study. The study was approved by the Ethics Committee Research of UZ/KU Leuven (S65328).

#### Data analysis

The group discussions were audio recorded and transcribed verbatim manually. The transcripts were analyzed according to the thematic framework with NVivo software (Release 1.3), using a combination of inductive and deductive coding [23]. The deductive coding was based on the findings of the first step of this study (i.e., the scoping literature review). The transcripts were analyzed by categorizing the relevant transcript sections to the thematic framework that was developed during the first step of this study. Additional codes that arose during the group discussions were added inductively to the coding tree. The group discussions also served to detect subcodes under the existing themes that were identified through the scoping literature review. Next, the results of the coding process were charted into a framework matrix and interpreted by the researchers. Ultimately, the results were narratively described, and illustrative quotes supporting the findings were extracted.

To guarantee the confidentiality of all participants, the recordings and transcripts were pseudonymized by removing all names and references towards the identifiable person (Cfr. Supplementary Material). These were replaced by a code randomly assigned to each participant per focus group, of which only the researchers have the code key to identify each participant.

The NGT also generates a small amount of quantitative data in addition to qualitative data. These are the result of the first and second ranking step (i.e., the five-point Likert scale) and are reported as an average score per determinant. The results of the first ranking step were presented to the participants, while the average scores per determinant of the second ranking were reported as the final result of this study (Cfr. Table 3). Prior to the study, a mean cutoff ranking score of 3.5 was defined by the researchers to identify the predominant determinants. The cutoff score was based on previous similar research, as well as on the perceptions of the NGT participants [27]. Finally, all average scores per determinant were ranked from high to low (Table 3).

## Results

### Scoping literature review

As discussed above, a scoping literature review was conducted prior to the focus group discussions in order to identify elements that drive prescribing decisions in general. These identified determinants were used as a starting point for the focus group discussions with Belgian physicians.

The predefined search strategy identified a total of 2587 records. After the removal of duplicates and screening of the title and abstract, 48 articles remained. Further screening of the full-text articles led to 10 articles being excluded. The literature search was updated at the time the researchers finalized the analysis, by again using the same search strategy to look for newly published articles. This resulted in an additional 15 records that were included in the qualitative analysis. Therefore, 53 articles were included in the final qualitative analysis. An overview of the literature search process, including the flow chart of the Preferred Reporting Items for Systematic Reviews and Meta-Analyses (PRISMA) [28], is included in the Supplementary Material.

Five main categories of determinants that drive prescribing choices were identified: (1) product-related, (2) physicians’ personal, (3) healthcare system-related, (4) patient-related, and (5) determinants related to the pharmaceutical company or brand. Each of these main categories was subdivided into specific determinants. An overview of all elements determining prescribing decisions with a brief explanation is provided in Table 1. A detailed overview of the different determinants of prescribing choices identified in each article can be found in the Supplementary Material, along with a summary of the key characteristics of each study (i.e., title, author, year, location, study objectives, design, and sample size).

**Table 1:** An overview of determinants driving prescribing decisions, as identified from the scoping literature review

Determinants of prescribing choices		Explanation
Product-related	Clinical data	Scientific clinical safety and efficacy data for a particular product.
	Cost	The cost for the respective medicinal product.
	Administration	The modalities regarding the administration of the product: route (i.e., subcutaneous, intravenous, oral), frequency, device, etc.
	Novelty	Whether the product was recently introduced to the market; prescribers wanting to be associated with new treatment approaches.
Physicians’ personal	Working environment	The setting in which the prescriber works (i.e., academic hospital, private hospital, general hospital, or private practice).
	Region	The region where a physician works within a country.
	Prescribing habits	Prescribers stick to the medicine that they have most experience with.
	Age	The age of the prescribing physician.
Healthcare system-related	Preference of key opinion leaders (KOLs)	Prescribing preferences of key opinion leaders (KOLs) or senior staff members.
	Clinical guidelines	The most recent scientific or clinical guidelines, or the lack of them.
	Time pressure	The limited time that they have available to discuss a new treatment with their patients.
	Financial incentives	Financial incentives developed by the government, or those related to the financing system of the hospital, etc.
Patient-related	Patient profile	Clinical characteristics of the patient, comorbidities, comedication, age, gender, lifestyle, etc.
	Patient preference	The preference of the patient for a particular product (or product characteristic) influences the prescribing decision.
	Knowledge or perception of patients	The perception that a patient may have regarding a particular medicine.
Related to the pharmaceutical company or brand	Marketing and promotion of the pharmaceutical company	Marketing or promotional activities of pharmaceutical companies including sponsorships, grants, value-added services, visits from pharmaceutical representatives, samples etc.
	Reputation (or trust) in the company or brand	The reputation of the pharmaceutical company that markets the medicinal product, or the reputation of a specific brand.

### Focus group discussions using the nominal group technique (NGT)

#### Demographics of participants

Nineteen physicians participated in the focus group discussions. Most participants were working in Flanders (*n* = 11), while the rest worked either in Wallonia (*n* = 6) or Brussels (*n* = 2). They included rheumatologists (*n* = 7), gastroenterologists (*n* = 6), dermatologists (*n* = 3), and endocrinologists (*n* = 3). All age groups were represented in this study, except for the youngest group of 18 to 29-year-olds. This was expected given the age at which physician specialists graduate in Belgium. Most of the physicians were working in an academic hospital setting (*n* = 10). A summary of the demographic characteristics of all participants can be found in Table 2.

**Table 2:** The demographics of the focus group participants

Participants’ demographics	N (%)
Age	
18–29 years	0 (%)
30–39 years	6 (32%)
40–49 years	5 (26%)
50–59 years	6 (32%)
60 years or more	2 (11%)
Region	
Flanders	11 (58%)
Wallonia	6 (32%)
Brussels	2 (11%)
Specialism	
Gastroenterology	6 (32%)
Dermatology	3 (16%)
Rheumatology	7 (37%)
Endocrinology	3 (16%)
Other	0 (0%)
Working environment	
Academic hospital	10 (53%)
General hospital	4 (21%)
Private hospital	2 (11%)
Private practice	3 (16%)
Other	
Total	19 (100%)

The results of the focus group discussions are presented below for each of the 17 determinants identified from the scoping literature review. An overview of all these elements, along with their average ranking after the second round, is summarized in Table 3.

**Table 3:** Illustrative quotes from participants during the focus group discussions, along with the average second round ranking scores

Determinants of prescribing choices	Illustrative quotes	Average ranking (1–5) ^b^
Product-related factors^a^		
Clinical data	“The newer insulins are insulins with a better action profile compared to traditional insulins. There are more clinical data showing that they are better, which is the main reason why physicians are prescribing newer insulins.” (FGD1_PHY3) “For many dermatologists, the lack of clinical trials for biosimilars is still a problem. The comparator trials between biosimilars and their originator are not there. Such trials are much more extensive in IBD and rheumatology.” (FGD3_PHY2) “In the beginning, there was a natural reluctance by most colleagues about biosimilars in terms of efficacy. Today, I think the concept of biosimilarity has become more and more known.” (FGD2_PHY4)	**3.6**
Cost	“In dermatology, more expensive and newer biologicals are being prescribed much faster because of their clinical benefits. That certainly outweighs the higher societal cost for most physicians.” (FGD1_PHY5) “If we look at the evolution and uptake of the newer group of IL-17 or IL-23 inhibitors, which is very high in dermatology, then it seems to me that we don’t care much about societal cost.” (FGD3_PHY2)	3.1
Administration	“The administration route is important for many physicians. I know that the uptake of JAK inhibitors within rheumatology is very high in Belgium. I suspect that has a lot to do with convenience for patients related to the oral administration.” (FGD3_PHY1)	2.6
Novelty	“It is always nice to work with new molecules. If you have the luxury of working in a university hospital, you get involved in clinical trials and you receive the opportunity to work with new products. However, I do not think that weighs heavily in the choice of a product.” (FGD1_PHY4)	2.2
Physicians’ personal factors		
Working environment	“Academic hospitals will switch to newer insulins somewhat more quickly because they are more likely to have gained earlier experience with these products in clinical studies compared to peripheral centers.” (FGD1_PHY3) “Working alone versus working in a hospital obviously influences your background knowledge regarding medicines. The working environment determines what kind of information you come in contact with. When you receive more information on a particular product, this definitely increases the chances you will prescribe it.” (FGD3_PHY3)	**3.9**
Region	“We have the impression that the more southern in Belgium the smoother prescriptions are made toward new biologicals and more expensive therapies.” (FGD1_PHY1) “If you look at JAK inhibitors, one product is mostly prescribed in Flanders, and a different product in Wallonia. There is no way we can explain that difference, unless a certain pharmaceutical representative is more active in one region than in the other.” (FGD3_PHY1)	**3.5**
Prescribing habits	“I would not overestimate the impact of prescribing habits. Only when it comes to biosimilars, it seems that we are reluctant to change. For new molecules, that does not seem to be the case.” (FGD4_PHY1) “It is in the pen of many physicians. In other words, many physicians will prescribe what one has experience with and be more conservative.” (FGD1_PHY1) “It often takes 10–15 years before you really see change in behavior because people are just very slow to change their habits, even if you are persuasive, even if you make guidelines.” (FGD2_PHY3)	3.4
Age	“There is a younger generation of rheumatologists that have never experienced the scarcity in therapeutic options. They are more likely to prescribe more expensive and new medications.” (FGD1_PHY1)	2.6
Healthcare system-related factors		
Preference of key opinion leaders (KOLs)	“If you get backup from colleagues or KOLs at the university centers, you are more likely going to follow their opinion as a physician. You become more at ease or confident to prescribe medicines KOLs are promoting.” (FGD4_PHY3)	**4.1**
Clinical guidelines	“There is very little information on biosimilars in the European guidelines. That could be improved.” (FGD3_PHY2) “Clinical guidelines play a role in the discussion between products with different MOAs, but they do not play a role at the product level. Therefore, they do not matter when making the choice to prescribe a biosimilar or not.” (FGD4_PHY4)	**3.5**
Time pressure	“Time is often used as an excuse to hide the real reason why physicians are not switching to biosimilars. [...] However, if you have to explain to a patient who has been in remission for 10 years that they have to switch to a biosimilar, that leads to problems. So yes, that does take time to explain.” (FGD3_PHY1) “As a dermatologist, you have very busy consultations and little time per patient. If you have to go convince a patient to make a switch to a biosimilar, that is just not feasible in that short time span.” (FGD1_PHY5)	3.4
Financial incentives	“I think incentives from the government do work. I hear that from colleagues as well. It is possibly because of the incentive that the click comes to prescribe biosimilars. Only then you are going to remove the barrier.” (FG1_PHY2) “The impact of the previous financial incentive in Belgium is very low. I would give 0 or 1 out of 5 on its impact. However, the impact of an appropriate incentive would be very high. There, I would give a 5.” (FGD4_PHY6)	3.0
Patient-related factors		
Patient profile	“I think we, as physicians, are convinced that patient profile and clinical data drive us above all else. However, in reality, that will be less the case than we hope.” (FGD2_PHY3) “The benefits of newer insulins will certainly weigh more heavily for patients with type 1 diabetes than for patients with type 2 diabetes. [...] In the type 2 diabetes group, I think we are a bit more conservative and stick to the older insulins. Newer insulins are used mainly for type 1 diabetes.” (FGD1_PHY3)	**3.8**
Patient preference	“It is remarkable that no patient has asked me so far whether they could switch a biosimilar? Not a single patient yet. They have already asked me to be prescribed the new IL-17 or − 23 inhibitor, though.” (FGD4_PHY3) “Patient preference is a difficult one, [...] most patients trust the physician or the practitioner. If you explain your choice well enough, patients will follow your decision. They are rarely going to come up with a suggestion of necessarily being treated with a specific product.” (FGD1_PHY4)	3.2
Knowledge or perception of patients	“There are patients who are very skeptical about biosimilars. For these patients, it is best not to prescribe them a biosimilar. You will lose too much time on that anyway.” (FGD4_PHY5) “I think knowledge and perception only really matters in a minority of patients. Patients mainly rely on what physicians think is best for them, much less what they have read or heard before about a medicine.” (FGD2_PHY1)	2.9
Factors related to the pharmaceutical company or brand		
Marketing and promotion of the pharmaceutical company	**General** “Physicians are reluctant to admit they are influenced by marketing and promotion. Physicians want to make a scientifically sound, patient-centered choice. You would rather not admit that you are influenced by marketing and promotion from the industry. Even though we all know that we are strongly influenced by it.” (FGD1_PHY1) **Value-added services** “We had a good agreement with the company that markets the originator biological about TDM. As a result, we could do dose optimization very quickly. These discussions with biosimilar companies were much more difficult, which has held back the uptake of biosimilars.” (FGD3_PHY3) **Visits by pharmaceutical companies** “Very often, it is the personal relationship with a pharmaceutical representative that makes prescribers stick with Humira.” (FGD4_PHY2) “The influence of pharmaceutical representatives in our field is not to be underestimated. Dermatologists are easily persuaded to take a certain therapeutic path.” (FGD3_PHY2) **Sponsorships or grants** “In many cases, pharmaceutical companies come up with an offer for a certain financial grant if you prescribe their product. This is in particular the case for biosimilar companies or the company marketing the originator. To be clear, companies are all doing this. They provide such things either as sponsorships or as a support for our hospital service.” (FGD3_PHY4)	**3.8**
Reputation (or trust) in the company or brand	“The reputation of the company will especially play a role when choosing between biosimilars, since different biosimilar manufacturers exist and not all of them have the same experience with biological medicines. You want to know what the supply chain is like or whether there are chances for shortages?” (FGD1_PHY1) “Quite some physicians tend to prefer companies that also invest in research and development. Companies that merely do copying are not preferred.” (FGD3_PHY2)	3.4

^a^ For each quote, the corresponding identification code is given of the respective participant who mentioned it. This code was assigned ad random to each participant per focus group

^b^ The average values refer to the results of the second ranking round. Each factor was ranked on a 5-point Likert scale, with following legend: 1 = very low impact, 2 = low impact, 3 = some impact, 4 = high impact, 5 = very high impact. Average values higher than 3.5 were underlined and highlighted in bold

#### Product-related

##### Clinical data

Physicians stated that the need for clinical data regarding biosimilars has evolved over the years. In the past, Belgian physicians had insufficient knowledge about the clinical development of biosimilars, which influenced their prescription practices. Until recently, uncertainty existed among Belgian physicians about the clinical differences when transitioning from an original biological to a biosimilar. These doubts are less prevalent nowadays due to an increase of positive experiences with biosimilars. It was agreed that when choosing between a biosimilar and its originator biological, doubts regarding reduced efficacy or safety are becoming less of an issue in Belgium. According to the participating physicians, the concept of extrapolation of indication does not pose an issue anymore for most Belgian physicians.

However, the situation differs when choosing between products with different mechanisms of action (MOA). In this case, the clinical data were considered important. For example, among the dermatologists, IL-17/23 inhibitors are considered clinically superior to existing off-patent alternatives (i.e., TNF-alpha inhibitors). This is one of the main reasons why existing off-patent biologicals are rarely prescribed for the treatment of new patients with psoriasis. This importance was also reflected in the average ranking score of 3.6 (Cfr. Table 3).

##### Cost

Physicians agreed that cost should be an important determinant, given the rising pharmaceutical expenditure in Belgium over the last few years. However, despite the important price differences between off-patent and competing patented products, it was believed that this only mattered for a small number of physicians when choosing a particular biological. The main reason for this would be that Belgian physicians are not sufficiently aware of the cost of medicines. Participants mentioned that there is a general lack of awareness about increasing pharmaceutical costs and the importance of a financially sustainable healthcare system. Moreover, the other determinants discussed in this article were believed to outweigh the impact of price considerations, according to the participating physicians.

According to the participants, cost considerations become an almost irrelevant determinant when choosing between an originator biological and its biosimilar(s) because of the limited price differences in the ambulatory care.

Overall, it was concluded that the cost of biological products has a limited influence on prescribing decisions in Belgium.

##### Administration

Physicians agreed that the administration modalities play a role for certain therapeutic areas. However, the influence of this depends greatly on the product or on whether different administration routes, devices, or frequencies are available. Off-patent biologicals in the ambulatory setting are always subcutaneously (SC) administered. Hence, the route of administration was considered to be unimportant. However, for certain therapeutic groups, oral (non-biological) alternatives are also available (e.g., JAK inhibitors). The oral administration route was thought to be one of the reasons for the current increase of JAK inhibitors being prescribed in Belgium, especially for patients with rheumatoid arthritis.

For most off-patent SC products, there are products available with different injection devices, frequencies of administration, and injection comfort. Physicians agreed that these differences, if present, play a role in the choice of a particular off-patent biological, although they are mostly related to patient comfort and patient preferences (see patient-related). Physicians concluded that administration modalities are certainly taken into consideration but should not be overestimated when looking at the overall picture.

##### Novelty

Notwithstanding the success of certain new alternatives (such as IL-17/23 inhibitors, patented TNF-alpha inhibitors, JAK inhibitors, etc.) compared to off-patent biologicals, the mere fact that these are new products was believed to only play a small part in prescribing choices. The other elements discussed in this study are more likely to be causing the observed shift in prescribing behavior.

#### Physicians’ personal

##### Working environment

Regarding the working environment of prescribers, the main point raised during the focus group discussions was that Belgian physicians working in hospitals or private practices differ in their access to information and certain support services. In academic hospitals, physicians would often have more access to information and are visited more frequently by pharmaceutical representatives. In addition, physicians in academic hospitals often already have experience with new MOA products through research studies, which would lead to a faster prescription uptake of these products. Differences in support services between individual hospitals, however, was considered to be even more influential by the participating physicians. Also, participants expressed that academic hospitals often have access to a larger nursing staff or more hospital beds. As a result, when pharmaceutical companies offer value-added services as a tool to promote their products to prescribers, this would influence their prescribing decisions to a lesser extent than for physicians working in general hospitals. Participants indicated that services provided by the pharmaceutical industry will have a lower impact on physicians working in private practices, which is particularly common among Belgian dermatologists and rheumatologists.

In general, physicians noted the importance of the prescriber’s working environment, but this difference was mostly linked to other determinants, such as pharmaceutical promotion, prescribing habits, and the influence of KOLs.

##### Region

Several physicians pointed out some demonstrable differences between Belgian regions. For example, in rheumatology the transition from existing off-patent TNF inhibitors to JAK inhibitors is more prominent in Wallonia. However, the participants could not provide a particular reason for this. In addition, it was also indicated that there is a difference in the use of SC-administered biosimilars between Flanders and Wallonia, with higher SC biosimilar prescription rates in Flanders. It was argued that such differences may be due to different pharmaceutical marketing approaches and prescribing habits in both regions.

##### Prescribing habits

Prescribing habits were described by physicians as one of the most important determinants of prescribing choices. It was confirmed across all specialties that physicians tend to stick with products they have experience with. As a result, originator biologicals are often preferred over biosimilars in Belgium as prescribers have had limited experiences with biosimilars in the ambulatory care to date. An associated fear of loss of clinical efficacy was believed to be the main underlying reason for this therapeutic inertia when making prescribing choices. However, participants mentioned that prescribing habits tend to be more influencial when choosing between reference products and biosimilars compared to when new MOA products are being considered. For the latter, physicians had the impression that the abovementioned therapeutic inertia plays less of a role as moves to new MOAs are common practice in the Belgian ambulatory care.

##### Age

Participating physicians agreed that the age of the prescribing physician plays little or no role in their prescribing choices. However, it was mentioned several times that older physicians, who have also known a time without the abundance of therapeutic options, are probably more inclined to prescribe the products that have been on the market for a longer period (i.e., off-patent products). Moreover, it was argued that younger physicians will have accumulated less experience with the more mature biologicals and are more likely to transition to new MOAs for this reason.

#### Healthcare system-related

##### Preference of key opinion leaders (KOL)

The preference of KOLs was regarded as a major determining element when making prescribing choices for off-patent biological medicines. Physicians indicated that when KOLs express a particular preference regarding biological medicines, this generally has an impact on the prescribing choices of their peers. However, the preferences of KOLs were said to be mostly shown for specific MOAs, and not for reference biologicals and their biosimilars. According to the participants, this might have resulted in the shift that has been seen from off-patent biologicals towards new MOA products. KOLs have been less outspoken about their preferences towards biosimilars. Physicians indicated that it is precisely because KOLs give little attention to biosimilars, fewer biosimilars are being prescribed.

##### Clinical guidelines

Regarding the impact of clinical guidelines as a determinant for choosing a biological, all physicians emphasized that there is a substantial difference between choosing a particular molecule versus an originator biological or biosimilar. Belgian guidelines are generally scarce, and so European guidelines are seen as impactful. Clinical guidelines were thought to be a substantial determinant when choosing between different MOAs and thus between different off-patent (or patented) biologicals. However, it was indicated that clinical guidelines usually do not influence the decision when choosing between an originator biological and its biosimilar. The main reason for this is that both at the Belgian and the European level, this choice is often not explicitly incorporated in the clinical guidelines.

##### Time pressure

There was agreement among the participating physicians that it takes time to sufficiently explain to patients a transition from an original biological to a biosimilar. It was indicated that the limited length of a consultation does not allow for enough time to provide an explanation of a possible switch to a biosimilar. However, it was emphasized that time constraints are a commonly used excuse not to prescribe biosimilars. Physicians from the various therapeutic areas admitted that time pressure only plays a minor role when choosing between an originator biological or biosimilar medicine. In reality, there are other more predominant elements that determine this choice.

Across the different therapeutic areas, there was an agreement that transitioning between different molecules was regarded as common practice without the concerns of time constraints. Hence, there is no reason to assume that this should be much different when transitioning to a biosimilar. Therefore, even if more explanation is required when transitioning to a biosimilar, this should not be a valid reason to avoid prescribing biosimilars.

##### Financial incentives

During the group discussions, it emerged that financial incentives can be an important determinant of prescribing choices for off-patent biologicals. It was pointed out that the right incentives to increase the use of biosimilars may have an important impact, although these are currently absent in Belgium. The financial incentive introduced in 2019 to promote biosimilar prescription was cited as an example of the negligible impact of individual financial incentives. An incentive that supports the quality of care could have a much larger impact according to the participants.

Moreover, the impact of an incentive to promote biosimilar prescription would differ between hospitals. As indicated earlier, the amount of support varies from hospital to hospital. Therefore, the potential impact of supporting incentives from the government is partly dependent on this. Physicians working in private practices indicated that financial incentives, whether on an individual basis or at the hospital level, have a minor impact on their prescribing choices.

#### Patient-related

##### Patient profile

Participating physicians agreed that the patient profile plays little or no role in the choice of an originator biological versus its biosimilar. When it comes to choosing between different molecules or MOAs, the patient profile does play a role. Comorbidities, age, lifestyle, socio-professional context, and comedication were mentioned as relevant examples in this context. Physicians indicated that these determinants, together with the administration modality, can largely determine medication adherence. For example, a less frequent administration would be more appropriate for an active or young patient. Participants also indicated that these aspects are often mentioned in clinical guidelines. Therefore, the importance patient profile is also linked to the availability of clinical guidelines.

##### Patient preference

Physicians indicated that the choice of a biological is only marginally influenced by the patient as most patients have limited preferences. If patients do have a preference, which is mostly for a particular injection device or administration route, it was mentioned that this obviously has a significant impact on the prescribing choice. Physicians confirmed that apart from a small group of patients, most patients generally have no preference regarding a biosimilar or originator biological.

##### Knowledge or perception of patients

Patient knowledge or perception about a particular type of medicine only has a minor effect on prescribing choices, as patients typically have a limited knowledge about biologicals. Physicians agreed that it may play a role with a small number of patients who have a rather negative perception towards biosimilar medicines. Nonetheless, if strong negative preconceptions about a particular medicine exists, this could have a significant impact on prescribing choices since these negative preconceptions may induce nocebo effects (i.e., the induction or exacerbation of symptoms through negative expectations about the therapy). Endocrinologists indicated that a resistance to taking insulin therapy can be an important barrier for patients with type 2 diabetes, as there is often an underlying perception of failure and shame when being treated with insulin.

#### Related to the pharmaceutical company or brand

##### Pharmaceutical marketing and promotion

The impact of promotion and marketing activities from the pharmaceutical industry was considered by all participating physicians to be very large. It was striking that the focus group participants mentioned that although its impact is undoubtably significant, physicians often deny this impact. The influence of everything related to pharmaceutical marketing was rated as especially high when it comes to prescribing an originator biological or its biosimilar(s). It was postulated that this is because physicians usually know that the originator biological and its biosimilar(s) are clinically equivalent products. Therefore, physicians acknowledged that marketing and promotion become more important determinants in this context, compared to when choosing between different molecules or MOAs where clinical differences might come into play.

Based on the suggestions provided during the focus group discussions, this category was divided into three subcategories: (a) value-added services, (b) visits by pharmaceutical companies, and (c) sponsorship or grants.

- **Value-added services**
  When choosing between an original biological or a biosimilar, the services that a company offers to physicians or hospitals were considered the most important determinant within the category of marketing and promotion. Examples cited by participating physicians included patient support programs, therapeutic drug monitoring (TDM), and nursing support. Companies offering such services are preferred when choosing between an original biological and its biosimilar(s). Usually, companies that market the originator biological offer these services already before biosimilars become available. Physicians, therefore, questioned why they would transition to a biosimilar if prescribers and patients would lose this important support. However, it was also indicated that physicians generally do not find this a transparent and correct system, and therefore, government incentives to support biosimilar prescribing remain important. As elaborated earlier, it became clear during the group discussions that the impact of these services also depends in part on the support already in place in the hospital where the prescriber works.
- **Visits by pharmaceutical companies**
  Physicians agreed that one should not underestimate the impact of representatives from the pharmaceutical industry. Consciously or unconsciously, prescribers will be influenced by this when choosing an off-patent biological. A good personal contact with the prescriber would certainly have an impact. Physicians pointed out that this was a reason why prescribers do not transition to a biosimilar, but also why they do transition more quickly to another MOA. After all, the alternative MOA products are often marketed by the same company as the originator biological, and thus, by the same pharmaceutical representative. The information provided by representatives during visits was also considered to have a great effect. The physicians indicated that they usually have little time to inform themselves about the latest products, and as a result, they are sometimes limited to the information provided to them by industry representatives.
- **Sponsorships or grants**
  Many pharmaceutical companies provide additional sponsorships to prescribers to conduct research with their pharmaceutical product. This was also mentioned as a common practice for biological medicines, both with innovator and off-patent biologicals. Physicians indicated that such sponsorships certainly increase the likelihood of prescribing these products. This may occur because the prescriber would like to give something back to the company. Also, the fact that the physician has already some experience with the product increases the likelihood of future prescriptions.

##### Reputation of the pharmaceutical company or brand

Participating physicians mentioned that the reputation and trust in the pharmaceutical company is an element that matters when choosing a medicine, but is certainly of less importance than promotional activities. When deciding between an original biological and a biosimilar, certain companies have a less favorable reputation among Belgian physicians than others. Participating physicians appear to prefer companies that also invest in drug research and development, rather than companies that purely produce or market biosimilars. Participants also indicated that prescribers are more likely to prefer biologicals produced by companies that have experience with producing biological products. Both elements were viewed by participants as reasons why biosimilars are less often considered in prescribing decisions. In general, it was assumed that reputation has an impact on prescribing behavior especially when choosing between products with the same MOA, such as between an originator biological and it biosimilar(s).

## Discussion

### Key study findings

This study identified five main categories of elements that may determine prescribing choices for off-patent biologicals in Belgium. These five categories were the result of a scoping literature review that looked for determinants of prescribing choices in general: (1) product-related, (2) physicians’ personal, (3) healthcare system-related, (4) patient-related, and (5) determinants related to the pharmaceutical company or brand. Each of these was subdivided into several subcategories, leading to a total of seventeen determinants. In the second phase of this study, focus group discussions using the NGT with Belgian physicians were conducted to validate these determinants for prescribing off-patent biologicals in Belgium. From this, seven elements emerged as predominant in this setting: clinical data, region, working environment, marketing and promotion, patient profile, clinical guidelines, and preference of KOLs.

One of the most discussed topics was the influence of pharmaceutical promotion. Physicians generally agreed during the group discussions that the influence of pharmaceutical promotion is substantial. Several reviews on the influence of pharmaceutical promotion have concluded as well that its impact on prescribing decisions is considerable [29–36]. This study confirms these findings also for off-patent biological medicines.

The other much debated element was the impact of the medication cost. Although not strongly reflected in the ranking scores, participating physicians appeared to agree that too little attention is paid to medication cost among Belgian prescribers. This was also demonstrated in a previous study of Belgian prescribing practices where shifts towards more expensive molecules rather than off-patent biologicals were observed [13].

It is clear that prescription choices are complex and determined by a wide range of elements [36]. This was illustrated by the considerable number of determinants that were identified from the literature, and the fact that almost all were considered important to some degree by the Belgian participants in the context of off-patent biological prescribing.

### Avenues for further research

This study, in addition to the existing knowledge [12–14], made an important step towards informing Belgian policymakers to create tailored measures for a more sustainable market for off-patent biologicals and biosimilars in Belgium. Nonetheless, in the future, a more thorough and quantitative examination of the determinants of prescribing behavior would be interesting. A quantitative approach with a behavioral model is therefore advisable. This qualitative study could serve as a base for creating such a behavioral model and for thoroughly investigating prescribing behavior in the future.

### Opportunities for policymakers

Investments should be made to continuously increase the awareness of physicians about the importance of cost-effective prescribing. In this regard, several opportunities for policymakers were listed in Table 4 based on the identified determinants of prescribing decisions for off-patent biologicals in Belgium.

**Table 4:** Overview of opportunities for Belgian policymakers based on the findings of this study

Category of determinants	Opportunities for Belgian policymakers
Product-related	• Make continued educational efforts for physicians to increase their awareness on cost-effectiveness and financial sustainability of healthcare • Provide guidance in the electronic prescribing software on cost-effective prescribing choices, by making use of alerts or offering alternatives when lower value medicines are chosen • Implement tailored prescribing targets (i.e., quota) for biosimilar medicines, in collaboration with the relevant scientific association(s)
Physicians’ personal	• Create tailored incentives (i.e., benefit-sharing) by considering differences in working environment, therapeutic area, and region
Healthcare system-related	• Stimulate KOLs to act as ambassadors for best-value biological medicines (including biosimilars), by involving scientific associations in policy discussions or decisions • Align reimbursement modalities with the most recent clinical guidelines • Reevaluate the reimbursement conditions regularly when the market situation changes upon loss of exclusivity or biosimilar market entry • Create tangible and tailored benefit-sharing incentives that support quality of care
Patient-related	• Involve patient associations from the relevant therapeutic domain(s) when designing policy interventions in order to incorporate the voice of patients in policy frameworks • Invest continuously in patient education on biological medicines with a focus on biosimilars, and disseminate information via Belgian patient associations
Related to the pharmaceutical company or brand	• Increase the transparency of pharmaceutical promotion that Belgian healthcare providers receive from the industry • Develop updated guidelines to physicians and industry about patient support programs or value-added services

Benefit-sharing incentives appear to have an influence on prescribing choices, although they will need to be introduced in the form of healthcare support within hospital departments and not as individual financial incentives at the level of the prescriber [37]. This study made clear that these needs differ between types of hospitals, regions, and therapeutic areas. Therefore, a certain amount of freedom on how hospital departments choose to deploy these incentives within their working environment is appropriate. More restrictive measures such as prescription targets (on biosimilars or on best-value biologicals) are helpful but should not be used as a standalone measure [27, 38, 39]. Invariably, prescribing targets should be accompanied by information, benefit-sharing initiatives, and other measures to empower physicians in their prescribing choices [40].

In an ideal world, resources are infinite and governments can provide all patients with access to the medicine with the best clinical outcomes or therapeutic value. However, in reality, budgets are limited and therefore the use of cost-effective therapies is essential [41]. In this way, clinical outcomes are maximized and as many patients as possible can be treated with the limited financial resources available [42, 43]. For this reason, clinical guidelines increasingly focus on the importance of cost-effectiveness in medicine selection [44–47]. The Belgian reimbursement committee usually adopts these clinical guidelines in their reimbursement modalities. However, in the specific case of JAK inhibitors versus TNF-alpha inhibitors in rheumatology, these have not yet been included in the reimbursement modalities. As a result, physicians are free to choose the various second-line products that are in competition with off-patent biologicals, regardless of the significant differences in treatment costs that have emerged after biosimilar market entry. The Belgian reimbursement committee should, therefore, periodically revise reimbursement modalities for off-patent biologicals as soon as they lose their market exclusivity or when biosimilars enter the market [10, 13]. This may result in bringing more cost-effective products into the treatment line sooner or at an earlier disease stage [48].

As mentioned above, pharmaceutical promotion may be an important determinant of prescribing decisions. Despite the existing regulations (i.e., Article 10 of the Belgian Law on Pharmaceutical products of March 1964 and Directive 2001/83/EC on Medicines for human use) and code of ethics (i.e., Mdeon) at the Belgian and European level [49–52], more guidelines on transparency and authorized promotion are desirable. Belgian physicians indicated that there is a need for more detailed transparency regarding what support physicians receive from pharmaceutical companies. This was also previously proposed by Medaxes, the umbrella association for the Belgian generic and biosimilar industry [53]. Moreover, in particular for value-added services and patient support programs, guidelines are advised on which services are allowed (and which are prohibited).

Given the rapidly evolving off-patent biologics landscape with new biological medicines losing their market exclusivities, new second-generation products, and new therapeutic classes coming to the market, it is important that policymakers act proactively. Policy measures should therefore be tailored to the product-specific context and implemented prior to the changing market environment when biosimilars enter the market.

### Study strengths and limitations

There have been no peer-reviewed research articles published that have examined prescribing choices for off-patent biologicals to date. Therefore, as far as the authors know, this study is the first to do so in an academic setting. By starting from elements found in the existing scientific literature and evaluating these further in the context of biological medicines with various Belgian physicians during focus group discussions, a comprehensive picture was obtained. The NGT was appropriate for this purpose as qualitative insights from the group discussions could be combined with a quantitative aspect to estimate the relative impact of each determinant in prescribing decisions.

This study sought to identify which aspects influence prescribing choices through a combination of a literature review and focus group discussions. The scoping literature review aimed to identify general determinants of prescribing choices from the scientific literature. Because of the broad search strategy and the fact that only one independent investigator performed the screening, relevant articles may have been missed (i.e., selection bias). However, the purpose of this scoping review was not to provide a complete overview of all articles describing determinants of prescribing behavior. Instead, the researchers aimed to give an overall picture of the existing literature on this topic, in order to allow for further exploration in a second step during focus group discussions.

A significant part of human decisions happens unconsciously, and people do not consciously make specific trade-offs all the time. This aspect is challenging to map through focus group discussions. In addition, we should also consider possible bias in the results because physicians may be more likely to name elements that should drive prescribing choices rather than those that actually drive their choices. However, this was avoided as much as possible by clearly informing the participating physicians about the research aims.

A disadvantage of the chosen qualitative method is that it is more difficult to draw conclusions about the entire population due to the relatively small sample. This selection bias is inherent to the purposeful sampling method [54]. Also, the majority of participants were active in academic hospitals. Considering the differences between academic hospitals and private practices or general hospitals, this may also have led to a certain bias in the results. Lastly, a generalization of the obtained conclusions to a wider geographical scope other than Belgium would not be appropriate.

As is inherent in qualitative research, personal biases and opinions may influence the interpretation of the research findings [55]. However, through close consultation with the researchers involved and a standardized protocol for each step of this study, the probabilities of this were kept to a minimum. In this way, reflexivity was embedded in the research process.

## Conclusion

Prescribing is inextricably linked to human choices and behavior, and thus should be understood in this context. Product-, patient-, healthcare system-, personal-, and pharmaceutical marketing-related elements play a role in prescribing decisions, underlining the complexity of prescribing choices. To increase the prescribing of best-value biologicals in the future, policy measures should be tailored to the product-specific context and implemented prior to the changing market environment when biosimilars enter the market.

## Supplementary Information

**Additional file 1.**

## Data Availability

The data generated or analyzed related to the literature review of this study are included in this published article and its supplementary information files. The datasets generated and/or analyzed during the focus group discussions are not publicly available due to privacy constraints but are available from the corresponding author on reasonable request.

## Abbreviations

NGT: Nominal Group Technique
KOL: Key Opinion Leader
NIHDI: National Institute for Health and Disability Insurance
IL: Interleukin
JAK: Janus Kinase
TNF: Tumor Necrosis Factor
MOA: Mechanism of Action
SC: Subcutaneous
TDM: Therapeutic Drug Monitoring

## References

[1] IQVIA (2021). The global use of medicines 2022: outlook to 2026.

[2] OECD (2022). Health spending (indicator).

[3] OECD (2022). Pharmaceutical spending (indicator).

[4] National Institute for Health and Disability Insurance (NIHDI) (2020). Budget 2021 – Begrotingsvoorstel van het Verzekeringscomité.

[5] IQVIA (2020). The Impact of Biosimilar Competition in Europe.

[6] OECD (2021). Health at a Glance.

[7] National Institute for Health and Disability Insurance (NIHDI) (2022). Budget 2022 – Begrotingsvoorstel van het Verzekeringscomité.

[8] European Medicines Agency (EMA) (2017). Biosimilars in the EU: Information guide for healthcare professionals.

[9] Cornes P, Bennett D (2018). Biosimilars.

[10] Simoens S, Vulto AG (2021). A health economic guide to market access of biosimilars. Expert Opin Biol Ther.

[11] Simoens S, Huys I (2022). Emerging insights into European Markets of Biologics, Including Biosimilars. Pharmaceuticals.

[12] Moorkens E, Vulto AG, Huys I, Vulto AG (2020). Biosimilars in Belgium : a proposal for a more competitive market. Acta Clin Belg.

[13] Vandenplas Y, Simoens S, Van Wilder P, Vulto AG, Huys I. Off-patent biological and biosimilar medicines in Belgium: a market landscape analysis. Front Pharmacol. 2021. 10.3389/fphar.2021.644187. 10.3389/fphar.2021.644187 PMC8091126 33953678

[14] Dylst P, Vulto A, Simoens S (2014). Barriers to the uptake of biosimilars and possible solutions: a Belgian case study. Pharmacoeconomics..

[15] HSE Medicines Management Programme (2019). Best-Value Biological Medicines: Tumour Necrosis Factor-α Inhibitors on the High Tech Drug Scheme.

[16] National Institute for Health and Disability Insurance (NIHDI) (2016). Convenant “Doorstart voor biosimilaire geneesmiddelen in Begië.”.

[17] National Institute for Health and Disability Insurance (NIHDI) (2019). Biosimilaire geneesmiddelen: Incentive voor het voorschrijven van biosimilaire geneesmiddelen buiten het ziekenhuis.

[18] Matjasko JL, Cawley JH, Baker-Goering MM, Yokum DV (2016). Applying behavioral economics to public health policy: illustrative examples and promising directions. Am J Prev Med.

[19] Connelly LB, Birch S (2020). Sustainability of publicly funded health care systems: what does Behavioural economics offer?. Pharmacoeconomics..

[20] Munn Z, Peters MDJ, Stern C, Tufanaru C, McArthur A, Aromataris E (2018). Systematic review or scoping review? Guidance for authors when choosing between a systematic or scoping review approach. BMC Med Res Methodol.

[21] Paré G, Trudel M-C, Jaana M, Kitsiou S (2015). Synthesizing information systems knowledge: a typology of literature reviews. Inf Manag.

[22] Qatar computing research institute (2022). Rayyan.

[23] Gale NK, Heath G, Cameron E (2013). Using the framework method for the analysis of qualitative data in multi-disciplinary health research. BMC Med Res Methodol.

[24] McMillan SS, King M, Tully MP (2016). How to use the nominal group and Delphi techniques. Int J Clin Pharm.

[25] Lago PP, Beruvides MG, Jian J-Y, Canto AM, Sandoval A, Taraban R (2007). Structuring group decision making in a web-based environment by using the nominal group technique. Comput Ind Eng.

[26] Palinkas LA, Horwitz SM, Green CA, Wisdom JP, Duan N, Hoagwood K (2016). Purposeful sampling for qualitative data collection and analysis in mixed method implementation research. Adm Policy Ment Health.

[27] Barbier L, Simoens S, Declerck P, Vulto AG, Huys I. Biosimilar use and switching in Belgium: avenues for integrated policy making. Front Pharmacol. 2022. 10.3389/fphar.2022.821616/. 10.3389/fphar.2022.821616 PMC9315422 35903323

[28] Page MJ, McKenzie JE, Bossuyt PM, Boutron I, Hoffmann TC, Mulrow CD (2021). The PRISMA 2020 statement: an updated guideline for reporting systematic reviews. BMJ..

[29] Hulscher MEJL, Grol RPTM, van der Meer JWM (2010). Antibiotic prescribing in hospitals: a social and behavioural scientific approach. Lancet Infect Dis.

[30] Teixeira Rodrigues A, Roque F, Falcão A, Figueiras A, Herdeiro MT (2013). Understanding physician antibiotic prescribing behaviour: a systematic review of qualitative studies. Int J Antimicrob Agents.

[31] Rose J, Crosbie M, Stewart A (2021). A qualitative literature review exploring the drivers influencing antibiotic over-prescribing by GPs in primary care and recommendations to reduce unnecessary prescribing. Perspect Public Health.

[32] Davari M, Khorasani E, Tigabu BM (2018). Factors influencing prescribing decisions of physicians: a review. Ethiop J Health Sci.

[33] Md Rezal RS, Hassali MA, Alrasheedy AA, Saleem F, Md Yusof FA, Godman B (2015). Physicians’ knowledge, perceptions and behaviour towards antibiotic prescribing: a systematic review of the literature. Expert Rev Anti-Infect Ther.

[34] Machowska A, Lundborg CS, Stålsby LC (2019). Drivers of irrational use of antibiotics in Europe. Int J Environ Res Public Health.

[35] Alowi M, Kani Y. Impact of pharmaceutical companies’ promotional tools on physicians’ prescription patterns: a systematic review. J Appl Pharm. 2018;10.

[36] Lublóy Á (2014). Factors affecting the uptake of new medicines: a systematic literature review. BMC Health Serv Res.

[37] Barcina Lacosta T, Vulto AG, Turcu-Stiolica A, Huys I, Simoens S (2022). Qualitative analysis of the design and implementation of benefit-sharing programs for biologics across Europe. BioDrugs.

[38] Remuzat C, Dorey J, Cristeau O, Ionescu D, Radiere G, Toumi M (2017). Key drivers for market penetration of biosimilars in Europe. J Mark access Heal policy.

[39] Moorkens E, Jonker-Exler C, Huys I, Declerck P, Simoens S, Vulto AG (2016). Overcoming barriers to the market access of biosimilars in the European union: The case of biosimilar monoclonal antibodies. Front Pharmacol.

[40] Barbier L, Simoens S, Vulto AG, Huys I (2020). European stakeholder learnings regarding Biosimilars: part II—improving biosimilar use in clinical practice. BioDrugs.

[41] Sculpher M (2000). Evaluating the cost-effectiveness of interventions designed to increase the utilization of evidence-based guidelines. Fam Pract.

[42] Chalkidou K, Glassman A, Marten R, Vega J, Teerawattananon Y, Tritasavit N (2016). Priority-setting for achieving universal health coverage. Bull World Health Organ.

[43] Bilinski A, Neumann P, Cohen J, Thorat T, McDaniel K, Salomon JA (2017). When cost-effective interventions are unaffordable: integrating cost-effectiveness and budget impact in priority setting for global health programs. PLoS Med.

[44] Smolen JS, Landewé RBM, Bijlsma JWJ, Burmester GR, Dougados M, Kerschbaumer A (2020). EULAR recommendations for the management of rheumatoid arthritis with synthetic and biological disease-modifying antirheumatic drugs: 2019 update. Ann Rheum Dis.

[45] Torres J, Bonovas S, Doherty G, Kucharzik T, Gisbert JP, Raine T (2020). ECCO guidelines on therapeutics in Crohn’s disease: medical treatment. J Crohn's Colitis.

[46] Gossec L, Smolen JS, Ramiro S, De Wit M, Cutolo M, Dougados M (2016). European league against rheumatism (EULAR) recommendations for the management of psoriatic arthritis with pharmacological therapies: 2015 update. Ann Rheum Dis.

[47] Davies MJ, Alessio DAD, Fradkin J, Kernan WN, Mathieu C, Management of hyperglycaemia in type 2 diabetes (2018). 2018 A consensus report by the American Diabetes Association (ADA) and the European Association for the Study of diabetes (EASD). Diabetologia.

[48] Dutta B, Huys I, Vulto AG, Simoens S. Identifying key benefits in European off-patent biologics and biosimilar markets: it is not only about Price! BioDrugs. 2020. 10.1007/s40259-019-00395-w. 10.1007/s40259-019-00395-w PMC7113204 31792843

[49] European Parliament. Directive 2001/83/EC On the Community Code relating to Medicinal Products for Human Use.

[50] Ministerie van Volksgezondheid en Leefmilieu (1964). 25 maart 1964 - Wet op de geneesmiddelen.

[51] MDeon (2018). Code voor Deontologie: Deontologisch Gezondheidsplatform.

[52] Federal Agency for Medicines and Health Products (FAMHP). Reclame, premies, voordelen, monsters. 2021. https://www.fagg-afmps.be/nl/MENSELIJK_gebruik/geneesmiddelen/geneesmiddelen/goed_gebruik_geneesmiddel/reclame. Accessed 8 Aug 2022.

[53] Medaxes (2019). Medaxes Memorandum 2019.

[54] Sharma G (2017). Pros and cons of different sampling techniques. Int J Appl Res.

[55] Guillemin M, Gillam L (2004). Ethics, reflexivity, and “ethically important moments” in research. Qual Inq.

