# Supplementary material for: Determinants of prescribing decisions for off-patent biological medicines in Belgium: a qualitative study
Source: BMC Health Serv Res. 2022 Sep 29;22:1211. doi: 10.1186/s12913-022-08591-1 (PMC9520107; doi:10.1186/s12913-022-08591-1)
Supplement: Supplementary file 1 — Additional file 1. [file 12913_2022_8591_MOESM1_ESM.docx]

**Supplementary Material**

**Determinants of prescribing decisions for off-patent biological medicines in Belgium: A qualitative study**

Yannick Vandenplas^1+^, Steven Simoens^1^, Philippe Van Wilder^2^, Arnold G. Vulto^1,3^, Florian Turk^4^, Isabelle Huys^1^

^1^KU Leuven, Department of Pharmaceutical and Pharmacological Sciences, Leuven, Belgium

^2^Ecole de Santé Publique, Université Libre de Bruxelles (ULB), Brussels, Belgium

^3^Hospital Pharmacy, Erasmus University Medical Center, Rotterdam, the Netherlands

^4^Unversity of Paderborn, Paderborn, Germany

^+^ Corresponding author: Yannick Vandenplas ([yannick.vandenplas@kuleuven.be](mailto:yannick.vandenplas@kuleuven.be))

**S1: Available biosimilar products in the Belgian ambulatory care setting (June 2022)**

| Molecule | Biosimilar product (brand names) | Date of reimbursement |
| --- | --- | --- |
| Adalimumab | Amgevita®  Imraldi®  Hyrimoz®  Hulio®  Idacio®  Yuflyma® | March 2017  August 2017  July 2018  September 2018  April 2019  September 2021 |
| Etanercept | Benepali®  Erelzi®  Nepexto® | January 2016  July 2019  February 2021 |
| Insulin glargine | Abasaglar® | June 2016 |
| Enoxaparin sodium | Ghemaxan® | January 2021 |
| Insulin lispro | None available in Belgium | |
| Teriparatide | None available in Belgium | |

**S2: Literature review protocol**

**Research question:**

What are elements that influence or determine prescribing decisions among physicians?

**Approach:**

A structured literature review on which factors or determinants have been identified in scientific literature regarding prescribing behavior or decisions in different contexts. Structured reviews and original research articles are both of interest.

**Databases:**

Pubmed (Medline), Embase

**Concepts:**

- Behavioral economics: Behavioral economics, nudging

- Healthcare and medicinal products/pharmaceuticals: Health, healthcare, pharmaceuticals, medicine

- Policy: Policy, intervention, measure

**Inclusion criteria:**

Full text available

Published between 2008 and 2021 (March 2021)

Only full text articles (no conference abstracts)

Scientific articles that discuss determinants of prescribing choices or behavior

Written in English

| **A: Prescribing**  **PUBMED** | | | **RES** |
| --- | --- | --- | --- |
| Concept | MeSH term | Free text (searched in title and abstract) | |
| Prescribing | “prescriptions” | Prescribing, prescription*, recipe | |
| Search query | ("Prescriptions"[Mesh] OR "prescribing"[Title/Abstract] OR "prescription*"[Title/Abstract] OR “recipe”[Title/Abstract]) | | |
| **B: Behavior** | | |  |
| Concept | MeSH term | Free text (searched in title and abstract) | |
| Behavior | “behavior” | Behavior*, behaviour* | |
| Search query | ("Behavior"[Mesh] OR "behavior*"[Title/Abstract] OR "behaviour*"[Title/Abstract]) | | |
| **C: Physician** | | |  |
| Concept | MeSH term | Free text (searched in title and abstract) | |
| Physician | “physicians” | Physician*, doctor* | |
| Search query | ("Physicians"[Mesh] OR "physician*"[Title/Abstract] OR "doctor*"[Title/Abstract] OR “practitioner”[Title/Abstract]) | | |
| **D: Decision, drivers** | | | |
| Concept | MeSH term | Free text (searched in title and abstract) | |
| Decision, driver | Decision making | Decision*, driver* | |
| Search query | (“decision making”[MeSH Terms] OR “driver*”[Title/Abstract] OR “decision*”[Title/abstract]) | | |

**Complete query:** ("Prescriptions"[MeSH Terms] OR "prescribing"[Title/Abstract] OR "prescription*"[Title/Abstract] OR “recipe”[Title/Abstract]) AND ("Behavior"[MeSH Terms] OR "behavior*"[Title/Abstract] OR "behaviour*"[Title/Abstract]) AND ("Physicians"[MeSH Terms] OR "physician*"[Title/Abstract] OR "doctor*"[Title/Abstract] OR “practitioner”[Title/Abstract])) AND (“Decision making”[MeSH Terms] OR “driver*”[Title/Abstract] OR “decision*”[Title/abstract])

**Results:** 696 (1/3/2021)

| **A: Prescribing** | | | **RES** |
| --- | --- | --- | --- |
| Concept | Emtree term | Free text (searched in title and abstract) | |
| Prescribing | Prescription | prescribing, prescription*, recipe | |
| Search query | ('prescription'/exp OR 'prescribing':ti,ab,kw OR 'prescription*':ti,ab,kw OR ‘recipe’:ti,ab,kw) | | |
| **B: Behavior** | | |  |
| Concept | Emtree term | Free text (searched in title and abstract) | |
| Behavior | Behavior | Behavior, behaviour | |
| Search query | ('behavior'/exp OR 'behavior':ti,ab,kw OR 'behaviour':ti,ab,kw) | | |
| **C: Physician** | | |  |
| Concept | Emtree term | Free text (searched in title and abstract) | |
| Physician | Physician | Physician*, doctor* | |
| Search query | ('physician'/exp OR 'physician*':ti,ab,kw OR 'doctor*':ti,ab,kw OR ‘practitioner’:ti,ab,kw) | | |
| **D: Decision, drivers** | | | |
| Concept | Emtree term | Free text (searched in title and abstract) | |
| Decision | Decision making | Decision* | |
| Driver |  | Driver* | |
| Search query | (‘decision making’/exp OR 'decision*':ti,ab,kw OR 'driver*':ti,ab,kw OR ‘decision making’:ti,ab,kw) | | |

**EMBASE**

**Search query:**

('prescription'/exp OR 'prescribing':ti,ab,kw OR 'prescription*':ti,ab,kw OR ‘recipe’:ti,ab,kw) AND ('behavior'/exp OR 'behavior':ti,ab,kw OR 'behaviour':ti,ab,kw) AND ('physician'/exp OR 'physician*':ti,ab,kw OR 'doctor*':ti,ab,kw OR ‘practitioner’:ti,ab,kw) AND (2008:py OR 2009:py OR 2010:py OR 2011:py OR 2012:py OR 2013:py OR 2014:py OR 2015:py OR 2016:py OR 2017:py OR 2018:py OR 2019:py OR 2020:py OR 2021:py) AND ('article'/it OR 'article in press'/it OR 'review'/it) AND (‘decision making’/exp OR 'decision*':ti,ab,kw OR 'driver*':ti,ab,kw OR ‘decision making’:ti,ab,kw)

**Results:**  1 891 (1/3/2021)

**S3: Literature review PRISMA flow diagram**

Additional records included through snowballing (n = 6) and additional search update (n = 9)

Records identified through database searching
(n = 2 587)

- PubMed (n = 640)
- Embase (n = 1947)

Records screened on title and abstract
(n = 2 072)

Duplicates removed
(n = 515)

## Identification

## Eligibility

## Included

## Screening

Full-text articles assessed for eligibility
(n = 48)

Studies included in qualitative synthesis
(n = 53)

## Records excluded (n = 2 024)

## Not relevant (n = 1 873)

## Wrong language (n = 34)

## No full text available (n = 19)

## Conference abstract (n = 22)

Too old (n = 76)

Full-text articles excluded
(n = 10)

**S3: Informed Consent Form (English), as approved by the Ethics Committee UZ/KU Leuven on May 21^st^, 2021**

**NIHDI project: Prescribing behavior of Belgian physicians regarding off-patent biological and biosimilar medicines**

Head of research: Professor Isabelle Huys

Clinical Pharmacology and Pharmacotherapy

KU Leuven

O&N II, Herestraat 49 – Box 521, 3000 Leuven

[Isabelle.huys@kuleuven.be](mailto:Isabelle.huys@kuleuven.be)

Tel.: +32 16 33 04 09 or +32 16 32 34 15

Contact person: Yannick Vandenplas

Clinical Pharmacology and Pharmacotherapy

KU Leuven

O&N II, Herestraat 49 – Box 521, 3000 Leuven

[Yannick.vandenplas@kuleuven.be](mailto:Yannick.vandenplas@kuleuven.be)

Tel.: +32 476 70 81 23 or +32 16 32 56 29

Dear Mr./Mrs.,

You are invited to voluntarily participate in a study which aims to identify prescribing drivers of Belgian physicians regarding off-patent biological and biosimilar medicines. Your insights about the different relevant aspects regarding prescribing behavior are very valuable to us from a Belgian policy point of view. Before you confirm to participate in this study, we ask you to read this information letter carefully. For any questions regarding the study, please contact the contact person mentioned at the top of this letter.

**What is the purpose of this study?**

During this study, we aim to identify drivers that influence prescribing choices of Belgian physicians for off-patent biological and biosimilar medicines. The ultimate goal is to have a better understanding about which factors influence physician’s decision making when prescribing off-patent biological or biosimilar medicines. Eventually, policy measures to stimulate the competition in the off-patent biologicals market can be adapted to these identified drivers.

**Who is conducting this study?**

This study is initiated by KU Leuven, in cooperation and with the support of the Belgian National Institute for Health and Disability Insurance (NIHDI). KU Leuven is responsible for the conduct of the study, i.e. group discussions, as well as for the processing of the collected data.

**Is this study scientifically and ethically justified?**

The Ethics Committee Research UZ / KU Leuven approved the study. Ethics committees verify if the rights of participants are respected by researchers during a study, if the balance between risks and benefits is beneficial for the participants and if the study is scientifically and ethically justified.

**Do you have to participate?**

Your participation is completely on a voluntary basis, and you have the right to refuse to participate in this study. If you decide not to participate, this will have no consequences. There is no cost associated with participating in this study and you will not receive any compensation to participate.

**What will be asked from you?**

We plan to conduct a group discussion, which will take approximately two hours. During a first preparation step, prior to the virtual group discussion, a short online questionnaire will also have to be completed. The group discussion will be held online (due to the current COVID-19 restrictions), so no transportation costs will be involved for participation in this study. The group discussion will be conducted in English. However, if required, participants will have the opportunity to express themselves in their mother tongue (Dutch or French). During the group discussion, several topics and related questions will be discussed related to prescribing behavior of Belgian physicians. A list of pre-identified drivers of prescribing choices will be prepared by the research team in advance, based on a scoping scientific literature review. The research team will ask you to grade these drivers, formulate possible additional drivers, and comment on the identified drivers. The group discussion will be audio-recorded and written out afterwards (ad verbatim).

**Do I have to participate?**

Your participation is completely voluntary. You can refuse to participate in the study. If you decide not to participate, this will have no consequences for you. There are no costs associated with participation in the study and no compensation is provided for participation. You have the right to withdraw your consent for any reason. You do not have to provide a reason for this. If you withdraw your consent, the data will be retained that were collected up to the time of your cancellation. This is to guarantee the validity of the study.

**How will your personal data be kept confidential?**

The group discussion will be audio recorded and do not include video recordings. In addition, a short online questionnaire will be conducted for demographic data about each participant and to grade the identified drivers. The audio recordings are written out (transcription), thereby names and organizations are pseudonymized. The collected data from the online questionnaires will be pseudonymized as well. In this way, the identity of the participants remains confidential everyone besides the head of research and contact person (Isabelle Huys and Yannick Vandenplas). Transcribing the group discussions will take place within three months after the group discussion has been conducted. To ensure confidentiality, only the head of research and contact person (Isabelle Huys and Yannick Vandenplas) team will have access to the key linking the code to the name of the participant, and to the recordings. In the final report, the researcher only uses pseudonymized written quotes obtained during the group discussion. During the study, the audio recordings and transcripts will be stored on a secured KU Leuven drive from the researcher. Destruction of the recordings and any personal notes of the researcher will take place after the completion of the transcription. The data collected during this study will be stored in a secure KU Leuven database. The confidentiality of the data is guaranteed in accordance with national and local data protection legislation, in particular the General Data Protection Regulation (GDPR).

**How will your coded (pseudonymized) data be used?**

Your coded data will be used to gain insights about gaps and solutions regarding the current situation of best-value biologicals in Belgium. The results will be analyzed and interpreted by researchers. The researchers have the intention to publish the coded results in a scientific journal, and possibly disseminate them via presentations at meetings or congresses.

**How long will your personal data be stored?**

After completion of the project, the head of research will further ensure safe storage of the transcripts and other files related to this study (i.e. audio recordings, online questionnaire results). After completion of the study all non-identifiable coded data will be transferred to a safe storage repository of the KU Leuven. In accordance with the KU Leuven policy, all files will be kept for maximum ten years from the end of the study.

**What rights do you have concerning your personal data?**

If you would like to review, correct, update, restrict, object to the processing or delete personal data, or if you would like to receive an electronic copy of the personal data you have provided, you can contact one of the persons mentioned at the top of this form. Your request for data deletion will be addressed within 30 days after your request has been confirmed. Such request may not be fulfilled in case that deletion renders or seriously impairs the study objectives. Please note that you may not be able to review some of the data until after the end of the study, and a request to delete your personal data cannot be fulfilled in case regulations and laws require your personal data to be retained. If you have questions about how we use data or wish to exercise your right to view, correct, and possibly stop further processing, you can always contact the researcher at the following contact address isabelle.huys@kuleuven.be or +32 16 33 04 09. If afterwards you still have special points of interest or wish to file a complaint, you can contact the KU Leuven privacy team at privacy@kuleuven.be. You can request the contact persons mentioned at the top of this form to forward any questions, concerns or complaints you may have to the data protection officer of the KU Leuven. You also have the right to lodge a complaint to the data protection authority in Belgium via e-mail: contact@apd-gba.be or phone: +32 (0)2 274 48 00.

**Please contact the contact person mentioned above for any questions regarding this study or to confirm your participation.**

**Thank you in advance for your interest and participation.**

Kind regards,

Yannick Vandenplas (PhD researcher)

On behalf of Prof. Isabelle Huys, Prof. Steven Simoens, Prof. Arnold G Vulto, Prof. Philippe Van Wilder, and Prof. Florian Turk

**Consent Form**

**Version 1.3 – 20210503**

**Please read the terms below. If you agree to these terms, please tick the box at**

**the bottom of this form to confirm your participation.**

- I have read the information letter regarding this study, and I have had the opportunity to ask questions or discuss any concerns about the study. In case of questions, they were clearly answered.
- I was given sufficient time to decide whether I am willing to participate in this study or not.
- I understand that data about me will be collected throughout my participation in this study and that the investigator and the sponsor of the study will guarantee the confidentiality of these data in accordance with applicable European and Belgian legislation.
- I am aware that participation in this study is completely voluntary and that the choice not to participate has no consequences. I am aware that I can decide to stop participating in this study at any time without any consequences.
- I give permission to the researchers to use the information gathered during this study (written notes, audio recordings, questionnaire results) for scientific purposes. All collected information will be processed pseudonymized (coded).
- I give permission that the coded data of this study can be used for publications in scientific journals.
- I give permission for my personal information to be stored for maximum 10 years. I am aware that such a request may not be fulfilled in case the information is already processed, deletion renders or seriously impairs the study objectives, or if regulations and laws that apply to this research require my personal data to be retained.
- I give permission that my coded data will be used to answer future research questions from researchers where the data will be processed in accordance with the European General Data Protection Regulation (GDPR) and the Belgian legislation on the protection of individuals with regard to processing of personal data. KU Leuven is responsible for the processing of my data.

**If you agree with the terms described above, please tick the box below to confirm your participation:**

I agree with all terms listed above and hereby confirm my participation in this project

Participant’s name Signature Date

Researcher’s name Signature Date

**S4: Overview of identified articles resulting from structured literature review**

| **Title (Author, year, location)** | **Objective, study design, sample size** | **Determinants of prescribing choices** |
| --- | --- | --- |
| Antibiotic prescription practices of pediatricians and pediatric residents in hospital care in Greece (Geitona, 2015, Greece) | Objective: Determine antibiotic prescribing practices in Greece  Study design: Cross-sectional questionnaire  Sample size: 275 physicians | Drug cost is not considered, gender, experience, type of hospital |
| Antibiotic prescribing in hospitals: a social and behavioural scientific approach (Hulscher, 2010, N/A) | Objective: Identify determinants that influence hospital usage of antibiotics  Study design: Literature review  Sample size: N/A | Cultural (international differences), type of hospital, socioeconomic factors (industry promotion/marketing, healthcare system), sociocultural (uncertainty) |
| Factors influencing the choice of new generation antipsychotic medication in the treatment of patients with schizophrenia (Edlinger, 2009, Austria) | Objective: Investigate the factors influencing the choice for an antipsychotic medicine  Study design: Prospective study  Sample size: 108 patients | Efficacy, safety profile, shared decision making |
| Understanding physician antibiotic prescribing behaviour: a systematic review of qualitative studies (Rodrigues, 2013, N/A) | Objective: Review qualitative studies focused on understanding physicians' perceptions of the factors, attitudes and knowledge influencing antibiotic prescription  Study design: Systematic literature review  Sample size: N/A | Clinical experience, ignorance, fear, confidence, indifference, complacency, responsibility of others, continuous medical education, university education, diagnostic uncertainty, time, signs of patient, anxiety of patient, symptoms, age of patient, clinical condition, economic and social factors, practice location of physician, policies, guidelines, group exposure influence, communication/organizational model, public health considerations, cost savings, financial incentives, pharmaceutical companies |
| The role of pharmaceutical marketing and other factors in prescribing decisions: The Yemeni experience (Al-Areefi, 2019, Yemen) | Objective: Explore different drivers of prescribing decisions and the role of pharmaceutical marketing in this process  Study design: Semi-structured interviews  Sample size: 32 physicians | Drug characteristics (dosage, form, cost, safety, efficacy), pharmaceutical company (marketing, image, advertisement), indications, patient preference or user friendliness, information, evidence, physician's experience |
| Why do physicians prescribe new antidiabetic drugs? A qualitative study in the Greek healthcare setting (Karampli, 2020, Greece) | Objective: Explore factors that influence the adoption of new antidiabetic medicines  Study design: Semi-structured interviews  Sample size: 10 physicians | Product related (relative advantage, risk, ease of use, cost, compatibility), prescriber related, patient related (clinical characteristics), regulatory measures, pharmaceutical industry (marketing, sponsorships), prescribing guidelines |
| Antibiotic prescribing in primary healthcare: Dominant factors and trade-offs in decision-making (Lum, 2018, Australia) | Objective: Identify factors influencing general practitioner’s antibiotic prescribing  Study design: Mixed methods (semi-structured interviews, followed by discrete choice experiment (DCE))  Sample size: 10 physicians (interviews) and 23 physicians (DCE) | Patient expectations/preferences, duration of symptoms, familiarity with patient, reassessment, life event |
| GPs’ approach to insulin prescribing in older patients: A qualitative study (Agarwal, 2008, Canada) | Objective: Explore rationale for prescribing decisions for diabetes mellitus (type 2) treatment  Study design: Semi-structured interviews  Sample size: 21 physicians (general practitioners) | Personal beliefs, intensiveness of current therapy |
| Factors influencing prescribing behaviour of physicians in Greece and Cyprus: results from a questionnaire-based survey (Theodorou, 2009, Greece/Cyprus) | Objective: Investigate attitudes and factors that influence prescribing decisions in Greece and Cyprus  Study design: Questionnaire  Sample size: 1204 Greek and 193 Cypriot physicians | Efficacy, delivery mode, dose, cost for the patient, patient preference |
| Factors influencing family physicians' drug prescribing behaviour in asthma management in primary care (Tan, 2009, Singapore) | Objective: Identify factors influencing prescribing decisions for asthma patients  Study design: Focus group discussions  Sample size: 29 physicians (general practitioners) | Uncertainty of diagnosis, patients’ beliefs, perceptions of patients, concerns about side effects, costs (consultation fees and medicine cost) |
| Intrinsic and external determinants of antibiotic prescribing: a multi-level path analysis of primary care prescriptions in Hubei, China (Liu, 2019, China) | Objective: Identify intrinsic and external determinants of antibiotic prescribing in primary care  Study design: Questionnaire and prescribing data analysis  Sample size: 499 physicians | - Intrinsic determinants: Knowledge, expertise, experience - External factors: Patient pressure, time pressure, financial incentives, colleagues; Institutional environments: area, socio economic setting |
| A qualitative literature review exploring the drivers influencing antibiotic over-prescribing by GPs in primary care and recommendations to reduce unnecessary prescribing (Rose, 2019, N/A) | Objective: Evaluate literature to determine drivers influencing over-prescribing of antibiotic prescribing in primary care  Study design: Literature review  Sample size: N/A | Physician factors (peers, experience, education, habits, lack of adherence to guidelines, laziness, fear of losing patients, diagnostic uncertainty, conflict aversion, concerns disease worsening), External factors (pharmaceutical industry, financial incentive, fear of complaints, lack of time, lack of shared decision making, lack of decision support, lack of clear guidelines), Patient factors (anxiety, patient pressure/demand, communication barriers, lack of education/understanding of patient, socio economic status, comorbidities) |
| Real-world evidence and the behavioral economics of physician prescribing (Feinberg, 2017, N/A) | Objective: Overview of literature on whether behavioral economic theories are relevant to prescribing decisions  Study design: Review of behavioral economic theories  Sample size: N/A | Heuristics, framing, default, loss aversion |
| Drivers of broad-spectrum antibiotic overuse across diverse hospital contexts: A qualitative study of prescribers in the UK, Sri Lanka and South Africa (Tarrant, 2021, United Kingdom/Sri Lanka/South Africa) | Objective: Map the multi-level influences of broad-spectrum antibiotic overuse across an international sample of high and low income, and public and private, hospital settings  Study design: Semi-structured interviews  Sample size: 46 physicians | Individual factors (uncertainty, risk reduction as a priority, perceptions as being the most effective, training/knowledge/experience, engagement with guidelines/policies), Social factors (social norms, clinical autonomy, hierarchy and colleagues), Structural factors (pressure, incentives, hospital environment, uncontrolled healthcare resources, patient poverty) |
| Doctors’ perspectives on the barriers to appropriate prescribing in older hospitalized patients: A qualitative study (Cullinan, 2014, Ireland and United Kingdom) | Objective: Identify why inappropriate prescribing occurs, which barriers exist, and which interventions are suitable  Study design: Semi-structured interviews  Sample size: 22 physicians | Environmental and context resources, knowledge, skills, social influences, memory/attention, decision processes. Barriers to behavioral change: An environment conducive to suboptimal prescribing (interruptions, lack of IT infrastructure, chaotic surroundings), strained resources (lack of targeted pharmacy input, poor collaboration between different levels of care due to lack of time and poor IT), lack of specific training (not enough education for undergraduates), poor patient education (poor knowledge of their own medication) |
| Understanding the determinants of antimicrobial prescribing within hospitals: the role of prescribing etiquette (Charani, 2013, United Kingdom) | Objective: Identify key determinants of antibiotic prescribing in hospitals  Study design: Semi-structured interviews  Sample size: 10 physicians, 10 pharmacists, and 19 nurses r midwives | Influence of senior physicians or KOL, local evidence-based policies |
| Factors influencing primary care physicians to prescribe antibiotics in Delhi India (Kotwani,2010, India) | Objective: Explore factors that determine antibiotic prescribing among primary care physicians  Study design: Focus group discussions  Sample size: 36 physicians | Diagnostic uncertainty, perceived expectation/demand from the patient, practice sustainability, financial considerations, medical sales reps influence, inadequate knowledge, lack of time, patient behavior, laxity with prescribing regulation |
| Determinants of physician antibiotic prescribing behavior: A 3 year cohort study in Portugal (Rodrigues, 2013, Portugal) | Objective: Assess the influence of determinants for antibiotic prescribing  Study design: Cohort study (questionnaires)  Sample size: 1094 physicians | Ignorance, complacency, responsibility of others, fear, indifference |
| Determinants of physicians' medication prescribing behaviour in primary care in Riyadh city, Saudi Arabia (Magzoub, 2011, Saudi Arabia) | Objective: Identify prescribing behavior determinants in primary care  Study design: Questionnaire  Sample size: 87 physicians | Sociodemographics, practice setting, continuing education, access to educational materials, pharmaceutical company representatives, patient factors |
| Social and professional influences on antimicrobial prescribing for doctors-in-training: a realist review (Papoutsi, 2017, N/A) | Objective: Elicit explanations behind antibiotic prescribing behaviors  Study design: Literature review  Sample size: N/A | Uncertainty, social and professional norms, hierarchy, fear of criticism |
| Understanding variation in primary medical care: a nine-country qualitative study of clinicians' accounts of the non-clinical factors that shape antibiotic prescribing decisions for lower respiratory tract infection (Brookes-Howell, 2012, Belgium/Spain/Italy/United Kingdom/the Netherlands/Hungary/Poland/Norway) | Objective: Investigate the non-clinical factors that determine antibiotic prescribing choices for lower respiratory tract infections  Study design: Semi-structured interviews  Sample size: 80 physicians (primary care) | Patient expectations, lack of treatment guidelines, physicians' characteristics (professional ethos, self-belief in decision making (uncertainty, confidence), commitment to shared decision making) |
| Antibiotic prescribing in long-term care facilities: A qualitative, multidisciplinary investigation (Fleming, 2014, Ireland) | Objective: Explore influencing factors and views of healthcare professionals regarding antibiotic prescribing in long-term care facilities  Study design: Semi-structured interviews  Sample size: 37 healthcare professionals (10 general practitioners, 4 consultants, 14 nurses, 9 pharmacists) | Knowledge, social influence, environmental context and resources (money, nurses support), beliefs about consequences, memory, attention, decision making |
| Why do general practitioners prescribe antibiotics for upper respiratory tract infections to meet patient expectations: A mixed methods study (Fletcher-Lartey, 2016, Australia) | Objective: Describe the role of patient expectations in antibiotic prescribing for upper respiratory tract infections  Study design: Questionnaire and semi-structured interviews  Sample size: 584 physicians (general practitioners) | Patient expectations, limited time, poor doctor-patient communication, diagnostic uncertainty |
| Factors influencing prescribing decisions of physicians: a review (Davari, 2018, N/A) | Objective: Review the literature on factors influencing prescribing decisions  Study design: Literature review  Sample size: N/A | Patient clinical characteristics, pharmaceutical industry, physicians’ attributes, patient preference, cost of the medicine |
| Physicians' knowledge, perceptions and behaviour towards antibiotic prescribing: A systematic review of the literature (Rezal, 2015, N/A) | Objective: Systematically review knowledge, perceptions and behavior related to antibiotic prescribing  Study design: Systematic literature review  Sample size: N/A | Patient expectations, severity and duration of illness, afraid of losing patients, economic factors, pharmaceutical company marketing, limited up-to-date information, inadequate knowledge, supply issues, healthcare infrastructure, financial incentives |
| Factors influencing deprescribing for residents in advanced care facilities: Insights from general practitioners in Australia and Sweden (Bolmsjö, 2016, Sweden/Australia) | Objective: Investigate behavioral factors influencing prescribing practices of general practitioners  Study design: Semi-structured interviews, focus group discussions, and literature review  Sample size: 72 participants (residents of long-term care facilities, relatives, staff members, general practitioners) | Intentions (know-how, evidence, norms or what KOLs do), skills & abilities (quality and quantity of human resources), environmental factors (healthcare system, financial incentives, teamwork) |
| Antibiotic prescribing in long-term care facilities: a meta synthesis of qualitative research (Fleming, 2015, N/A) | Objective: Review the findings of qualitative studies examining factors that influence prescribing decisions of antibiotics in long-term care facilities  Study design: Systematic literature review  Sample size: N/A | Knowledge, prescribing practices, social factors (interactions between HCPs/residents families) |
| Drivers of irrational use of antibiotics in Europe (Machowska, 2019, N/A) | Objective: Review the evidence regarding the main drivers of antibiotic prescribing  Study design: Literature review  Sample size: N/A | Lack of public knowledge and awareness, access to antibiotics without prescription, leftover antibiotics, lack of adequate education, pharmaceutical promotion, diagnostic uncertainty, patient-doctor interaction) |
| Main factors affecting physicians' prescribing decisions: The Iranian experience (Sharifnia, 2018, Iran) | Objective: Evaluate the associations between influenced factors and drug prescriptions of Iranian physicians  Study design: Questionnaire  Sample size: 460 physicians | Payment type (insurance, managed care), patient factors (age, race, gender, comorbidity, treatment history), product's characteristics (cost, safety, efficacy, side effects) |
| Factors influencing hematologists-oncologists prescription of chemotherapy drugs in cancer treatment in Iran: An interview questionnaire study (Abolfazil, 2017, Iran) | Objective: Investigate the influencing factors for hematologists-oncologists’ prescribing of chemotherapeutics  Study design: Interviews and questionnaire  Sample size: 10 physicians (oncologists) | Product properties (clinical effectiveness, price, quality, popularity, side effects, beliefs about generics using facility), pharmaceutical marketing (company image, availability, country name of manufacturer), scientific/experimental confirmation (clinical trials/evidence, scientific journals, sponsorship for conferences, EMA/FDA approval, prescribing guidelines) |
| New medicines in primary care: A review of influences on general practitioner prescribing (Mason, 2008, N/A) | Objective: Review the determinants of uptake, influence of geographics, price, cost, and financial incentives on prescribing behavior  Study design: Literature review  Sample size: N/A | Product (safety, efficacy, cost), financial incentives, patient-doctor relationship, structural/environmental issues (patient expectations) |
| Generic medicines: Greek physicians’ perceptions and prescribing practices (Tsiantou, 2009, Greece) | Objective: Investigate the prescribing profile of physicians and identify factors influencing generic prescribing  Study design: Questionnaire  Sample size: 1204 physicians | Preference for branded products, opinion on generics' efficacy and safety, physicians' age, patients' insurance coverage, patients' income, medicine cost |
| Influence of pharmaceutical company engagement activities on the decision to prescribe: A pilot survey of UK rare disease medicine prescribers (Jandhyala, 2020, United Kingdom) | Objective: Investigate the influence of pharmaceutical non-promotional activities on prescribing habits  Study design: Questionnaire  Sample size: 34 physicians | (Non-) personal engagement activities of pharma companies (e.g., scientific meetings, pharmaceutical visits, advisory boards, etc) |
| The influence of information, brand, medical representatives and sales promotion on physician prescribing decision (Murshid, 2018, Yemen) | Objective: Determine the influence of marketing activities on prescribing decisions  Study design: Questionnaire  Sample size: 393 physicians | Brand of the product |
| Factors influencing antibiotic prescribing decisions among inpatient physicians: a qualitative investigation (Livorsi, 2015, United States) | Objective: Examine the influence of professional and psychosocial factors on prescribing behavior in the inpatient setting  Study design: Semi-structured interviews  Sample size: 30 physicians | Diagnostic uncertainty, behavior of peers/supervising staff (hierarchy) |
| Provider decisions to treat respiratory illnesses with antibiotics: Insights from a randomized controlled trial (Branche, 2016, United States) | Objective: Evaluate factors influencing antibiotic prescribing for lower respiratory tract illness  Study design: Questionnaire  Sample size: 134 physicians | Patient factors (age, expectations, medicolegal concerns, clinical conditions) |
| Factors involved in making decision to prescribe medications for psychiatric disorders by psychiatrists: A survey study (Rajendran, 2012, United States) | Objective: Identify factors that influence prescribing decisions for psychiatric disorders  Study design: Questionnaire  Sample size: 26 physicians | Patient factors (symptoms, severity, diagnostic categories), past experience with medication, side effect profile, patient and family's preference |
| Impact of pharmaceutical companies' promotional tools on physicians' prescription patterns: A systematic review (Kani, 2018, N/A) | Objective: Review the evidence on the influence of pharmaceutical promotional activities on prescribing decisions  Study design: Systematic literature review  Sample size: N/A | Promotional tools (meetings with reps, gifts, samples, pharmaceutical promotional events) |
| Knowledge is power: General practitioners’ prescribing of new oral anticoagulants in Ireland (Murphy, 2018, Ireland) | Objective: Investigate factors determining new oral anticoagulant (NOAC) prescribing  Study design: Questionnaire  Sample size: 221 physicians | Practice size, location (region), previous experience with medicine, drug interactions, efficacy, monitoring requirements, patient non-compliance, co-morbidities, cost, socio-economics status, administrative burden, patient preference, local and national prescribing guidelines, side effects |
| The complex phenomenon of dysrational antibiotics prescribing decisions in German primary healthcare: A qualitative interview study using dual process theory (Poss-Doering, 2020, Germany) | Objective: Investigate factors influencing non-rational antibiotic prescribing decisions  Study design: Semi-structured interviews  Sample size: 27 physicians (primary care) | Uncertainty (diagnosis), prognosis, continuity of care, patient expectations, educate efforts help to overcome, guidelines/recommendations, incentives/resources, primary care networks |
| Factors that influence rheumatologists' anti-tumor necrosis factor alpha prescribing decision: a qualitative study (Gavan, 2019, United Kingdom) | Objective: Investigate factors determining the decision to prescribe TNF-alpha inhibitors  Study design: Semi-structured interviews  Sample size: 11 physicians (rheumatologists) | External environment influences (NICE recommendations, clinical commissioning groups, cost pressures, published clinical evidence, colleagues in different hospitals, pharmaceutical industry), internal hospital influences (systems to promote compliance with clinical recommendations, internal treatment pathways, hospital culture), individual-level influences (patient influence, clinical autonomy, consultant experience, perception of DAS-28 outcome) |
| Determinants of antibiotic prescribing for upper respiratory tract infections in an emergency department with good primary care access: a qualitative analysis (Chan, 2019, Singapore) | Objective: Elucidate determinants of antibiotic prescribing in emergency departments  Study design: Semi-structured interviews  Sample size: 9 physicians | Reliance on clinical knowledge and judgement, patient related factors (uncertainty avoidance, patient profiles), patient-physician relationship (patient demand, time constraints, conflict of interest), perceived practice norms, policies and treatment guidelines, patient education and awareness |
| Uncertainty as a critical determinant of antibiotic prescribing in patient with an asthma exacerbation: a qualitative study (Stefan, 2020, United States) | Objective: Identify factors influencing antibiotic prescribing in patients with asthma exacerbations in the hospital  Study design: Semi-structured interviews  Sample size: 16 physicians | Diagnostic uncertainty, awareness/agreement with existing guidelines, confidence in their ability to guidelines in challenging cases, perceived risk of patient deterioration without treatment, fear of litigation, habit and clinical inertia, prescribing within the group, lack of information of antibiotic prescribing rates, lack of time/resources. Four main themes: emotional, behavioral regulation, knowledge, beliefs |
| A national cross-sectional study on socio-behavioural factors that influence physicians' decisions to begin antimicrobial therapy (Velasco, 2011, Germany) | Objective: Assess which factors determine antimicrobial prescribing decisions  Study design: Questionnaire  Sample size: 3 492 physicians | Patient factors (age, diagnosis, clinical condition), personal factors physician (region, age, sex), pharmaceutical companies, experience with failed therapy, uncertainty |
| Systematic review of factors associated with antibiotic prescribing for respiratory tract infections (McKay, 2016, N/A) | Objective: Review evidence investigating factors associated with antibiotic prescribing for respiratory tract infections  Study design: Systematic literature review  Sample size: N/A | Patient level (sex, age, medical comorbidities), physician level (specialty, perceptions about medicine), area level (geographic location, experience) |
| Determinants associated with doctors’ prescribing behaviors in public hospitals in China (Pan, 2021, China) | Objective: Identify determinants of prescribing behavior of physicians  Study design: Questionnaire  Sample size: 444 physicians | Behavioral attitude, subjective norms, perceived behavioral control, |
| Factors, perceptions and beliefs associated with inappropriate antibiotic prescribing in German primary dental care: A qualitative study (Böhmer, 2021, Germany) | Objective: Identify barriers and facilitators for prudent usage of antibiotics in dental care  Study design: Semi-structured interviews and focus group discussions  Sample size: 18 physicians (9 participants for the interviews and 9 for the focus group discussions) | Uncertainty, lack of competence, guidelines, lack of empowerment, colleague physicians, patient demand, medico-legal concerns (liability) |
| The opioid-prescribing practices of Australian general practice registrars: An interview study (Prathivadi, 2021, Australia) | Objective: Understand and examine the factors influencing prescribing decisions for opioid medication  Study design: Semi-structured interviews  Sample size: 20 physicians (general practitioners) | Poor support and supervision, lack of confidence in de medicine (or medicine class), safety (struggle to translate prescribing guidelines into practice) |
| Inappropriate antibiotic prescribing: Understanding clinicians’ perceptions to enable changes in prescribing practices (Laka, 2021, Australia) | Objective: Identify perceived barriers to appropriate antibiotic prescribing  Study design: Questionnaire  Sample size: 180 physicians | Diagnostic uncertainty, access to guidelines and prescribing information, clinical experience, healthcare setting (hospital versus primary care), patient expectations |
| Factors associated with the decision to prescribe and administer antipsychotics for older people with delirium: A qualitative descriptive study (Tomlinson, 2021, Australia) | Objective: Explore factors associated with antipsychotic prescribing for older people with delirium  Study design: Semi-structured interviews and focus group discussions  Sample size: 42 healthcare providers (25 nurses and 17 physicians) | Safety, a last resort, workload, dilemma (difficult medication choices), anticipating to worsening of condition, influence of peers/colleagues |
| What influences prescribing decisions in a multimorbidity and polypharmacy context on the acute medical unit? An interprofessional, qualitative study (Rivers, 2021, United Kingdom) | Objective: Understand prescribing decisions in the context of hospital acute admissions unite  Study design: Focus group discussions  Sample size: 39 physicians and 9 pharmacists | Patient characteristics (age, pathology, family, adherence), medicine characteristics (clinical picture, avoiding harm, polypharmacy, guidelines, iatrogenesis, medication review), pharmacist factors (expertise, checker and optimizer role), trustworthiness (distrust of information source), marketing effects, reliability of medication history, competing priorities (time, pressure for take home-prescribing), responsibilities of prescribers (reluctance to change, passing the buck) |
| What influences healthcare providers’ prescribing decisions? Results from a national survey (Price, 2021, United States) | Objective: Examine the influence of pharmaceutical promotions and contact with industry on prescribing decisions  Study design: Questionnaire  Sample size: 2000 healthcare providers (1300 physicians, 350 physician assistants, and 350 nurses) | Pharmaceutical promotion, contact with pharmaceutical industry, colleagues, key opinion leaders |
| Determinants of antibiotic over-prescribing for upper respiratory tract infections in an emergency department with good primary care access: a quantitative analysis (Huang, 2021, Singapore) | Objective: Investigate the factors influencing prescribing decisions for antibiotics in emergency care patients with upper respiratory tract infections  Study design: Questionnaire  Sample size: 130 physicians | Place of medical education, perceived compliance with prescribing practices in the department, perceived over-prescribing in the department, clinical symptoms, diagnostic uncertainty, clinical imaging |
